# Analyzing the impact of fintech industry and green financing on energy poverty in the European countries

**DOI:** 10.1016/j.heliyon.2024.e27532

**Published:** 2024-03-09

**Authors:** Zeenat Zia, Ruoyu Zhong, Muhammad Waqas Akbar

**Affiliations:** a College of Economics, Shenzhen University, Guangdong, 518060, China; b China Center for Special Economic Zone Research, Shenzhen University, Guangdong, 518060, China

**Keywords:** Energy poverty, Fintech industry, Energy efficiency, Green finance

## Abstract

In the fourth industrial revolution, the fintech has significantly expanded during the last several years, and this has caused scholars to worry about how much electricity is being used. Because energy poverty is one of the most critical social policy concerns facing the majority of nations in the world in the modern era. This study adds to what has already been written by looking at how the fintech industry affects the environment and energy in European countries. The current study investigates how the growing awareness of the need to preserve energy and the environment has an effect on society, and analyzes the role of the fintech industry, green finance, energy efficiency, and research and development on energy poverty across European nations from 2013 to 2020. To estimate long- and short-term impacts, DOLS and FMOLS are used along with diagnostic tests. The outcomes found that there is a tight relationship between energy poverty and all the factors taken into consideration (fintech, green finance, energy efficiency, and R&D). EU governments should employ “green finance" to encourage and enable the fintech industry since fintech plays a vital role in enhancing environmental effectiveness. The financing of environmentally friendly projects is very beneficial and might help alleviate energy poverty. The findings also indicate that more financing, ecological subsidies, and social assistance programs are necessary in order to satisfy the needs for energy and put an end to energy poverty in Europe. Policymakers in the tech world may be especially interested in the results.

## 1 Introduction

One of the main drivers advancing social and economic growth is energy. The current global energy system views energy poverty as a serious economic challenge. In 2010, the International Energy Agency (IEA) reported that 1.6 billion people were living in energy poverty. By 2030, almost 1.4 billion fewer individuals will be able to obtain power. But if technology advances quickly enough, it may help overcome the constraints imposed by the scarcity of natural resources and promote sustainable economic development. The reduction of high energy consumption development is made possible by the advanced technological advancement and innovation. The fourth industrial revolution was sparked by advanced technological advancement in the previous 30 years (4IR). These technologies—such as big data, blockchain, and the internet of things—have produced the information required for green finance and will ultimately aid in the growth of the digital economy.

Thakor [1] delved into the far-reaching consequences of advanced trading in areas such as cryptocurrencies, payment systems, insurance, and credit within the realm of blockchain digital markets. Shahbaz et al. [2] emphasized the significant influence of technical advancements such as digital banking, internet shopping, 3D printing, and electric cars in transforming people's lives worldwide. Consequently, it can be inferred that this paradigm shift is poised to usher in a digital era for our economic and financial systems. “Fintech,” short for financial technology, is a term used to describe the innovative application of technology, particularly software and digital platforms, to deliver financial services and streamline financial transactions. It encompasses a broad spectrum of activities, including mobile banking, online payments, peer-to-peer lending, blockchain-based cryptocurrencies, and other digital financial innovations. Fintech has had a profound impact on how individuals and businesses manage their finances, making financial services more accessible, efficient, and convenient.

**Fig. 1:**
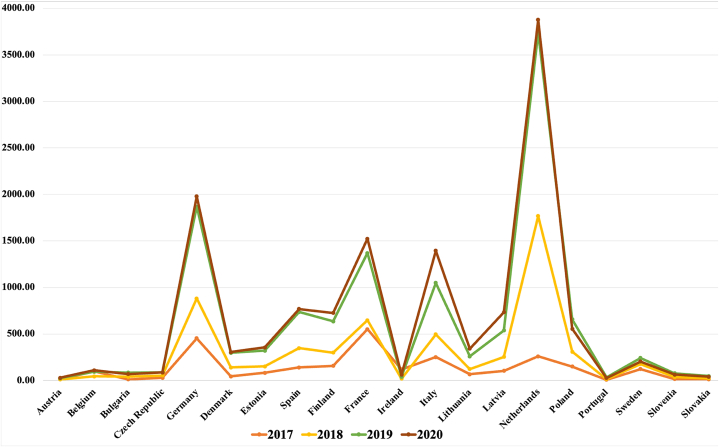
Fintech credit US$ mn (Source: WDI 2022).

Fintech's fast expansion seeks to diverge from conventional financial practices, resulting in the development of novel business structures and their implementation to enhance financial services in countries [3]. In 2018, the Fintech sector received investments totaling 112 billion dollars, according to Zverkov et al. (2019). The investment in financial technologies by European countries has increased for a better future of their economies, as shown in Fig. 1. The graph compares the fintech credit (US$) from 2017 to 2020 and it can be seen clearly that fintech credit has been increased in 2019 and 2020 as compared to previous years and it will be a sharp increase in coming years.

Nowadays, fintech has changed how global economies see their financial landscapes [4]. There is a dearth of literature on the relationship between financial technology and the environment, despite the abundance of studies explaining the connection between financial technology and economic growth and financial development. Yet, a lot of scholars are looking at how financial technology and the environment are related by examining financial products like cryptocurrency. Muhammad et al. [5] used four effects—direct, wealth, business, and substitution—to explain the connection between the fintech industry and the environment. However, Sadorsky [6], and Çoban and Topcu [7] also looked into these effects. Electric products with higher demands for electricity and energy consumption, such as phones, mobile devices, and other electric products, now have access thanks to the fintech industry, these goods directly degrade the environment's quality. Additionally, the fintech industry's cryptocurrency sector is thought to be electricity-intensive. Because cryptocurrencies require more electricity and use more energy, there are more carbon emissions and environmental risks. Malone (2014), De Vries [8] claims that the energy consumption of cryptocurrencies alone is equivalent to that of economies such as Austria and Ireland. According to Gangwar et al. [9], this produces a substantial quantity of electronic trash that is harmful to the environment. Sarwar et al [10] investigated the determinants of electricity consumption and accurate electricity price forecasting, with a particular focus on the eastern region of Saudi Arabia. Surprisingly, temperature is found to be a less relevant predictor of electricity consumption in this region, contrary to prior research. In the eastern region, electricity price exhibits a negative association with consumption. Machine learning techniques, especially the support vector machine, demonstrate superior predictability compared to traditional methods. Furthermore, the support vector machine is employed to forecast carbon emissions trends linked to electricity consumption. The study's findings hold significant policy implications, providing valuable insights for policymakers dealing with electricity consumption determinants and price forecasting.

Qin et al [11] explored the impact of fintech innovations on the environment. It introduces an advanced green environmental index that incorporates environmental, economic, resource, and financial indicators, a novel approach. Fintech is found to have a significant, positive relationship with the green environmental index, with financial breadth, depth, and digitalization playing key roles. The impact of fintech is consistent across different Chinese regions. Additionally, fintech partially mediates the influence of green credit and investment on the green environmental index. This research suggests promoting fintech innovation and green financing adoption as potential policy measures. Another research by Aziz et al [12] investigated the impact of environmental technology, environmental taxes, and renewable energy consumption on ecological footprints in East Asia from 1999 to 2019. It focuses on Japan, North Korea, South Korea, Mongolia, and China. Environmental technology emerges as a pivotal factor, showing a significant negative correlation with ecological footprints, indicating its potential for reducing environmental impacts. In contrast, the influence of environmental taxes and renewable energy consumption appears more limited. Urban population growth and economic development exacerbate ecological footprints. These findings emphasize the crucial role of technological innovations in environmental conservation and suggest a need to reevaluate policy priorities in East Asia. This research provides valuable insights for stakeholders, advocating the utilization of environmental technology while reconsidering existing policy tools.

Additionally, according to Lee et al. [13] and Katz et al. [14] it has improved the effectiveness of banking and financial systems overall, which has helped banks perform better. According to reports, financial technologies are positive externalities of quick financial innovation and financial inclusion. Based to research by Bollaert et al. [15], more investment is required to meet the objectives of the Paris Agreement and the Sustainable Development Goals (SDGs). Investments in financial technologies like the Internet of Things, blockchain, and big data would therefore have a greater potential to advance our understanding of green finance. In order to attain the objectives set forth in the Paris Agreement and SDGs, there is a compelling need for increased investments. Specifically, investments in cutting-edge financial technologies, e. g blockchain, internet of things and big data hold substantial promise in enhancing our comprehension of green finance. “Green finance.” often referred to as sustainable finance, is a concept that pertains to financial products and services designed to support environmentally sustainable and socially responsible initiatives. These initiatives typically aim to reduce the environmental footprint and promote sustainable practices, such as investments in renewable energy, energy-efficient technologies, and projects that mitigate climate change and environmental degradation.

A pioneer in block chain technology is Europe. Fintech and blockchain are required for low-carbon, climate-resilient investing in order to attain the SDGs. Little study and empirical analysis on the link between fintech and the environment is currently accessible in the literature. Fintech helps cut GHG emissions, which benefits the environment, according to a few studies, including Tao et al [16] investigation of the topic. Despite this, Muganyi et al. [17] discovered a connection between environmental harm and green finance and the fintech industry. The empirical results indicate that the fintech industry and green financing are critical to a sustainable environment. Nevertheless, researchers, such as Wang et al [18] and Liu and Song [19] looked into the relation among CO_2_ emission and financial development and they found that financial development tip to a rise in emissions.

**Fig. 2:**
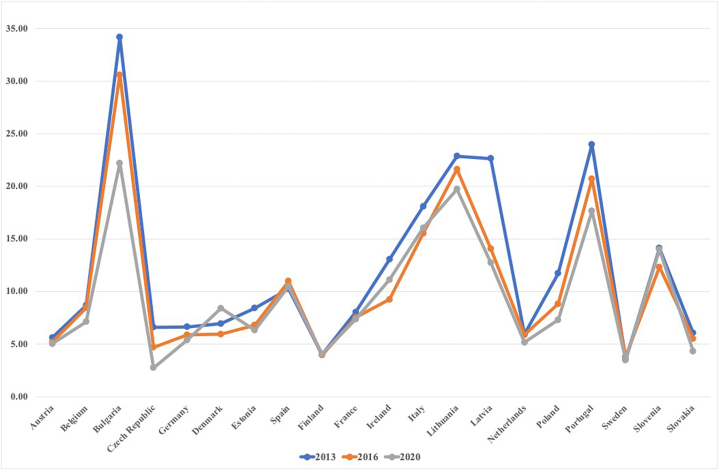
Energy poverty in European countries (Index generated by following [21])).

According to research by Chien et al. [20] green fiscal policies have a big influence on lowering energy poverty through improving energy efficiency. The fintech industry, energy poverty, and the environment are interconnected and act as double-edged swords. From the litrature it is ralised that as the techionolgy is improving the availability of energy is also very imperative to use that technology. And most of the countries in the world are faciong the issue of energy poverty. Fig. 2 explains, the energy poverty index, the figure above clearly represents that the higher the energy poverty index the higher rate of energy poverty will be in the country.

One advantage of the technology revolution in financial services and economic systems is that it may lead to greater environmental quality. However, technological advancement may also have a detrimental effect on environmental quality because of the growth in energy consumption, which also leads to an increase in electronic waste [16]. Energy poverty was the unintended result of this, energy is a crucial input component for economic activities that support economic development. The fourth scientific and technological revolution, together with financial developments, has begun in the global economy. Like the Internet of Things, block chains, and big data have a greater potential to further our understanding of green finance.

**Fig. 3:**
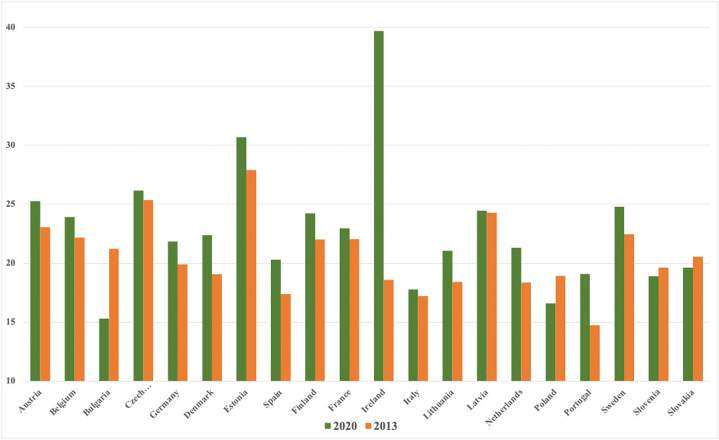
Purchase of pollution control equipment and plants million euro (Source [23]).

Qu and Hao [22] investigated how the internet economy may help China's energy poverty issue. The empirical results show that digital innovation contributes significantly to the decrease of energy poverty. Financial development may be a measure for lowering energy poverty by increasing investment in green technology innovation and increasing money flow into clean energy-related businesses. Innovation in financial technology facilitates the transition of the global economy to reduced greenhouse gas levels. Investment in environmental preservation initiatives is just as important as fintech. European countries have implemented a number of pollution-reduction measures, such as trading carbon emissions, boosting the use of renewable energy sources, and tightening environmental regulations. Muhammad et al [5] empirically analysis that financial technologies are becoming more environmentally friendly by using energy-saving and green technology. Financial innovations also lessen reliance on conventional energy-intensive businesses, which eventually increases per capita GDP and environmental efficiency. Fig. 3 displays statistics from European nations on investments made between 2013 and 2020 in machinery and plants for pollution control (also known as overall environmental protection activities). On the right-hand side, the bars in orange depict the data in the year 2013 whereas, the bars in green shows the data in the year 2020.

In European countries the green financing also improves environmental effectiveness and functions as a green equipment replacement strategy. It can be a payment for the installation of renewable energy systems and environmentally friendly plants. Because of fintech, there is less conversation on energy security and poverty (financial technologies). Nonetheless, a lot of research covered the connection between environmental concerns and energy consumption. We discovered that there is still much to be learned about the empirical relationship between energy poverty, energy security, and climate change (CO_2_ emissions) in the context of the fourth industrial revolution (Fintech).

The originality of our current research lies in its examination of how investments in fintech may contribute to reducing energy poverty and addressing environmental challenges, thereby boosting environmental protection activities within the European Union (EU). Our study's core objectives encompass investigating the fintech sector's impact on environment and energy poverty in EU. With the aim of advancing future economies, our research incorporates various factors, including energy efficiency, green financing, R&D, FDI and GDP. It is worth noting that our study delves into the multifaceted effects of financial technologies, particularly fintech investments, in alleviating energy poverty through the promotion of green financing and cutting-edge technology development. These fintech-based enterprises require substantial energy for the functioning of economic and financial systems. Our theoretical framework delves into the intricate relationships between green finance, fintech, R&D, energy efficiency, and FDI, collectively influencing energy poverty. We aim to comprehend how green finance and fintech innovations can enhance affordability and energy access, ultimately mitigating energy poverty. Strategies focused on energy efficiency and R&D play a pivotal role in optimizing resource utilization and improving energy access. Additionally, FDI acts as a catalyst for technology transfer and the sustainable development of energy infrastructure. Through our theoretical framework, we endeavor to shed light on the complex dynamics of energy poverty, providing original insights into the collaborative mitigation of its impact.

## 2 Materials and methods

### 2.1 Data and description of variables

This study aims to analyze the role of fintech industry, green financing, energy efficiency, R&D, GDP, and FDI in reducing energy poverty. Data of 20 European countries from 2013 to 2020 have been taken in this study based on data availability. The restricted data availability, especially for the fintech industry—which is still relatively young and lacks historical data—led to the selection of the 20 EU nations and the shorter time period. Fintech data is taken from the Cornelli et al. [24] database, which has been extensively used in the literature by several environmental and finance researchers, including Carbó-Valverde et al. [25], Kowalewski and Pisany [26], and Papadimitri et al. [27]. Measured in US dollars, fintech value represents a nation's overall fintech credit for a given year. Index of energy poverty has been calculated by following the research of Rodriguez-Alvarez et al. [21]. Whereas investment in plants and environmentally friendly equipment for pollution is measured as green finance. On the positive side, it provides a straightforward and precise definition of green finance, which enhances the clarity and aligns with widely accepted terminology in the field. However, it may be excessively narrow, focusing primarily on pollution control equipment, while green finance typically encompasses a broader array of environmentally friendly investments, such as renewable energy, sustainable infrastructure, and more. This specificity could potentially restrict the scope of what is considered green finance and might exclude other sustainability-driven investments. Furthermore, this definition simplifies the multifaceted nature of green finance, overlooking its intricacies and diverse applications. The study's data covers a time span from 2013 to 2020, encompassing eight years of information for 20 European countries. However, several limitations should be acknowledged.

The analysis is constrained by data availability, particularly in fintech, which is comparatively novel and lacks historical data. The study's definition of green finance focuses primarily on pollution control equipment, potentially limiting the scope and excluding other environmentally friendly investments. The reliance on data sources like Eurostat and the World Bank ensures credibility but may introduce variations in data quality and consistency across countries. Nevertheless, the study provides valuable insights into the role of fintech, green financing, and related factors in reducing energy poverty, offering transparency regarding data sources and variables. The World Bank (2022) and Eurostat [23] are the sources of the data. Table 1 displays the description of the variable, and Table 2 displays the descriptive statistics of the variables utilized in the econometric study.

**Table 1:** Variables description.

Variables	Description	Abbreviations	Source
Energy Poverty	Index generated by following [21]	EP	WDI
Fin Tech	Fintech is taken as credit USD million	FT	WDI
Green Finance	Total investment in activities related to environmental protection (equipment and plant million euro)	GF	Eurostat
Energy Efficiency	GDP (PPP constant 2017 per kilogram of oil equivalent) per unit of energy consumption	EE	WDI
FDI	GDP proportion of foreign direct investment expressed in million euros	FDI	WDI
GDP	GDP per capita	GDP	WDI
R&D	GDP share of research and development expressed in million euros	RD	Eurostat

**Table 2:** Descriptive statistics.

	EP	FIN	GF	EE	RD	FDI	GDP
**Mean**	10.385	226.346	21.681	12.583	1.865	3.397	35195.205
**Median**	7.675	48.480	21.220	11.749	1.695	2.747	33814.064
**Std. Dev**	6.820	501.219	4.401	3.885	0.867	11.281	17716.178
**Minimum**	2.757	0.010	14.750	6.386	0.440	−40.863	7143.462
**Maximum**	34.175	3742.920	53.590	25.359	3.410	81.302	82744.148
**Obs**	160	160	160	160	160	160	160
**EP**	1.000						
**FIN**	−0.174	1.000					
**GF**	−0.350	−0.018	1.000				
**EE**	0.082	0.074	0.127	1.000			
**RD**	−0.616	0.133	0.150	−0.018	1.000		
**FDI**	0.035	−0.277	−0.078	0.154	−0.131	1.000	
**GDP**	−0.551	0.205	0.417	0.523	0.707	0.026	1.000

### 2.2 The specification of econometric model

Globalization has made factor omitting, residual interdependency, and cross-sectional dependence major issues in panel data research in the current age. Therefore, because of the unobserved shocks that are included into the residuals are most likely to result in considerable dependence in panel models and results in inaccurate and misleading conclusions, we start with checking cross sectional dependency [28]. The null hypothesis in the cross-sectional dependence test is that there is no cross-sectional dependency, whereas the alternative hypothesis is that there is cross-sectional reliance. The alternative hypothesis in the cross-sectional dependence test is the existence of cross-sectional dependency, whereas the null hypothesis is the lack of cross-sectional reliance.

The variables' degree of integration was then examined using cross-sectionally dependent unit root tests (CADF and CIPS), since the presence of a unit root may lead to problems like incorrect results. Prior to the estimate of the econometric model, the identification of long-run linkages among the variables under consideration is crucial. Hence, we used the residual tests for cointegration developed by Pedroni [29] and Kao [30]. DOLS and FMOLS are employed in order to assess the long-term impacts. Compared to OLS, FMOLS and DOLS perform better, and they are useful for endogeneity and autocorrelation [31,32]. Adopting the following functional form of the model allows for the analysis of panel data while accounting for the effects of time and nation ([33]; Yuelan et al., 2021). A heterogeneous panel causality test is used to get the findings about causation for European economies (Dumitrescu and Hurlin 2012).

In panel data analysis, the Cross-Sectional Dependency Test is utilized to detect potential interdependencies among cross-sectional units within the dataset. This test serves to unveil whether unobservable factors or common shocks affect multiple units simultaneously, which can lead to biased parameter estimates and inaccurate conclusions. The purpose of the CIPS (Cross-Sectional Augmented Phillips-Perron) and CADF (Cross-Sectionally Augmented Dickey-Fuller) tests is to evaluate the panel's time series data integration state. These tests assume that individual time series should either be stationary or share a common stochastic trend, a critical consideration when studying panel data. For long-term relationship analysis, the DOLS (Dynamic Ordinary Least Squares) and FMOLS (Fully Modified Ordinary Least Squares) models come into play. They both rely on the assumption of cointegration among the variables, meaning they exhibit a stable, long-run relationship. These models also address endogeneity issues, with FMOLS further enhancing efficiency. These tools collectively aid researchers in addressing challenges related to cross-sectional dependence, non-stationarity, and long-term relationships, ensuring robust and precise results in panel data analysis.

The functional form of the model is given below in eq (1):

(1) EP_it_ = f (FT_it_, GF_it,_ EE_it_, R&D_it_, FDI_it_, GDP_it_, U_it_)

The estimated model's econometric form is as follows:

(2) EP_it_ = δi+ λi + β1_i_FT_it_+ β2_i_GF_it_ + β3_i_EE_it_ + β4_i_R&D_it_ + β5_i_FDI_it_+ β6_i_GDP_it_+μ_it_

In equation (2), the explicit EU nation are shown by *“i”*, and the time period is shown by “*t”*. In the presented model, λ_i_ and δ_i_ represent trends and particular nation impacts, respectively, whereas *β*_*1*_ *to β*_*6*_ represent the impact size of energy efficiency, fintech, green finance, R&D, FDI, and GDP.

## 3 Results and discussion

### 3.1 Descriptive analysis

Results of statistical descriptive analysis and correlation matrix of all variables are shown in Table 2. Consequently, the statistical properties of the variables are consistent for estimation. The matrix of correlation shows a potential projected relationship between energy poverty and the considered variables. Mean, median, stranded deviation, minimum values, and maximum values are given in the table below. The correlation matrix indicated that the correlation between all the independent variables is approximately from 0.1 to 0.3. If there is little correlation between the variables, multicollinearity will be less of an issue.

In Table 3 the results of cross-sectional dependency of each variable are showed, which shows that p-values are less than 0.05 for all the considered variables. As a result, it was determined that the null hypothesis is not true and that cross-sectional dependency exists. According to statistics from the Breusch-Pegan LM test, several variables in panel data exhibit cross-sectional reliance among EU economies.

**Table 3:**
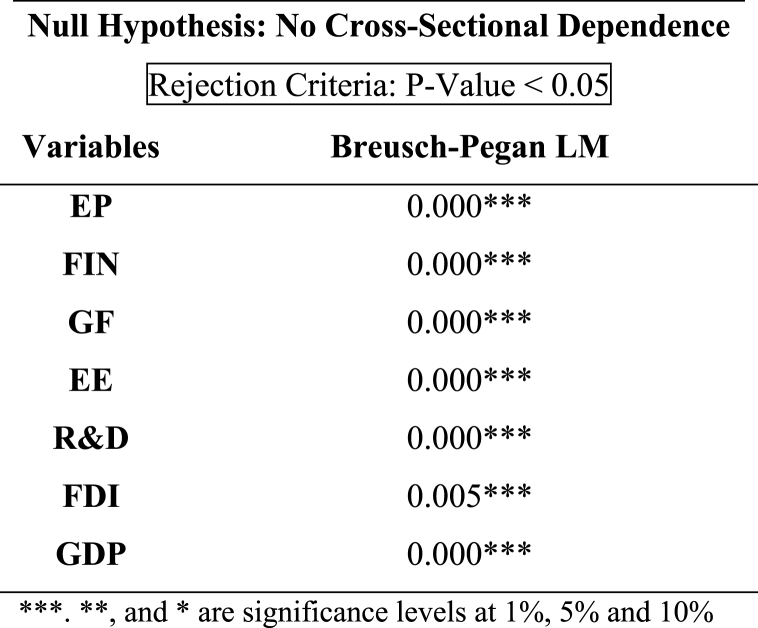
Results of Cross-Section Dependence.

After confirming that there is cross-sectional dependence in the data, In the presence of cross-sectional dependency, we have produced dependable and consistent findings using the CADF and CIPS unit root tests [34]. Unit root results are shown in Table 4, confirming the integrated level of all the variables and the outcomes showed that all of the variables are integrated of order I (0) since the p-value is lower than 0.05. The next stage is to conduct co-integration tests to examine the long-term relationship. In this research, Pedroni [29] and Kao [30] were utilized, and the residual co-integration test was also used to the model to demonstrate the existence of a long-term link among the variables. The findings are shown in Table 4.

**Table 4:** Results of unit-root.

Variables	CADF		CIPS	
	Value	Decision	Value	Decision
**EP**	3.980**	At level	−1.817*	At level
**EE**	3.482***	At level	−1.836**	At level
**FDI**	−2.557***	At level	−3.483***	At level
**FIN**	−3.951***	At level	−1.881*	At level
**GDP**	−19.78*	At level	−2.381**	At level
**GF**	5.054***	At level	−1.379*	At level
**R&D**	1.345*	I (0)	−1.865*	I (0)

Note: ***. **, and * are significance levels at 1%, 5% and 10%.

After the unit root test, it is time to determine the long-term connection. To do so, Kao residual co-integration and Pedroni cointegration tests are utilized. To ascertain long-term connections between variables, the Pedroni panel cointegration test (1999) employs seven statistical measures. These statistics include three group statistics (rho, ADF, and PP) as well as panel v, panel PP, panel rho, and panel ADF metrics. Table 5 displays Pedroni panel cointegration and Kao residual co-integration results. Since most statistical findings must be significant at 1%, 5%, or 10%, the results demonstrate the rejection of the null hypothesis, no cointegration. Energy poverty, fintech, green finance, energy efficiency, research and development, FDI, and GDP in EU nations have a long-term link. The results of p-values and t-stat showed out of seven, six values are statistically significant, explaining the long run relation.

**Table 5:**
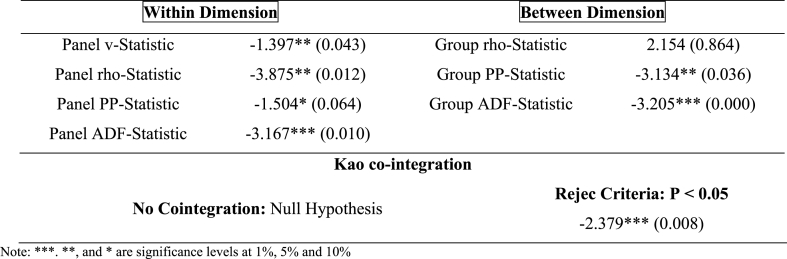
Pedroni and Kao co-integration results.

### 3.2 Empirical results of FMOLS and DOLS

We proceed to the cointegration regression analysis since the aforementioned tests demonstrate that there is an association between the variables in the long run. Verifying the existence of a long-term relationship is the only objective of the cointegration test. Thus, to evaluate the dynamics and kind of causation for the panel, this research used FMOLS and DOLS. Table 6's results demonstrated a statistically significant negative link between fintech and energy poverty. As financial technologies grow, energy poverty will decrease, according to the fintech coefficient. The values from FMOLS and DOLS are negative 0.001 and negative 0.771, indicating that there is more financing available for fintech to expand, Liu et al [35] found the same in their study. According to FMOLS and DOLS, this will result in a reduction of energy poverty of 0.1% and 77%, respectively. According to the findings of green financing, an increase in green financing would result in a 1 percent drop in energy poverty according to FMOLS and a 39 percent decrease in energy poverty according to DOLS, the results are in line with Zhao et al [36], Zhang et al [37]. Energy efficiency and energy poverty have a negative relationship, therefore for FMOLS and DOLS, respectively, a 1% improvement in energy efficiency would result in a 111 and a 78% reduction in energy poverty. According to FMOLS and DOLS, the coefficient of R&D shows a negative correlation with energy poverty, with values of −2.144 and −3.85 respectively. Yet, at a 1% level, FDI and GDP show positive and statistically significant outcomes, Li et al [38] had similar result.

**Table 6:** Results of FMOLS and DOLS.

Variables	FMOLS		DOLS	
	Coefficient	P-Value	Coefficient	P-Value
**FIN**	−0.001	0.047**	−0.771	0.089*
**GF**	−0.103	0.054*	−0.395	0.009***
**EE**	−1.118	0.005***	−0.784	0.000***
**R&D**	−2.144	0.093*	−3.385	0.000***
**FDI**	0.011	0.537	0.037	0.197
**GDP**	0.010	0.000***	0.412	0.038**

Note: ***. **, and * are significance levels at 1%, 5% and 10%.

### 3.3 Results of panel DH causality test

The aforementioned findings support the long-term connections between the variables, although they fall short of establishing a causal link. In order to resolve this problem and identify the causality among green finance, fintech, R&D, energy efficiency and energy poverty for European economies, a heterogeneous panel causality test is used. The Granger causality test served as the foundation for the development of this test by Dumitrescu and Hurlin. By utilizing Z-bar stats and W-bar stats, it also solves the problem of heterogeneity.

The findings presented in Table 7 shed light on the intricate relationships between various key variables. Notably, fintech, green finance, and energy poverty exhibit statistically significant values, indicating strong associations between these factors. These correlations suggest the presence of a bidirectional relationship between these variables, implying that changes in one can influence changes in the others, and vice versa. The p-values offer further insights into causality. They reveal that energy poverty is influenced by energy efficiency and R&D efforts, indicating a causal effect on energy poverty from these factors. However, it's important to clarify that this establishes a one-way causation, where improvements in energy efficiency and increased R&D investment cause a reduction in energy poverty, but not vice versa.

**Table 7:** Results of DH causality.

Null Hypothesis	w-bar stat	z-bar stat	p-value	Decision
FT ≠ cause EP EP ≠ cause FT	4.81 5.62	2.86 3.04	0.003*** 0.002***	Bidirectional causality FT ↔ EP
GF≠ cause EP EP ≠ cause GF	8.14 3.13	4.38 3.02	0.000*** 0.069*	Bidirectional causality GF→ EP
EE ≠ cause EP EP ≠ cause EE	5.41 2.54	2.78 0.10	0.028** 0.888	Unidirectional causality EE → EP
R&D ≠ cause EP EP ≠ cause R&D	12.10 1.12	9.68 −1.75	0.000*** 0.240	Unidirectional causality R&D → EP
FDI ≠ cause EP EP ≠ cause FDI	4.61 5.81	1.75 2.80	0.094* 0.005***	Bidirectional causality FDI ↔ EP
GDP ≠ cause EP EP ≠ cause GDP	4.16 5.31	1.65 2.10	0.094* 0.005***	Bidirectional causality GDP↔ EP

Note: ***. **, and * are significance levels at 1%, 5% and 10%.

Additionally, the results highlight a bidirectional relationship between GDP and foreign direct investment (FDI), indicating mutual causation between these economic indicators. This suggests that changes in GDP can influence FDI, and changes in FDI can, in turn, affect GDP. One noteworthy finding is the negative impact of fintech on energy poverty. The substantial and negative fintech coefficient suggests that an increase in fintech utilization leads to a reduction in energy poverty. However, it's essential to emphasize that while this points to a strong correlation, it does not establish a direct causal relationship. This effect can be attributed to the fintech sector's role in promoting the adoption of green and energy-efficient technologies, ultimately assisting businesses in reducing their energy consumption and dependence on conventional energy sources.

These findings align with the study conducted by Bizo (2019), who demonstrated that innovative solutions like Amazon Web Services (AWS) have the potential to substantially reduce carbon emissions within the information technology sector, by as much as 88%. AWS's energy-efficient practices outperform outdated IT systems by a factor of three, illustrating the transformative power of technology in environmental conservation. The development of fintech within the green industry is further bolstered by the European Environmental Regulation Authority, which promotes sustainable practices, ultimately steering the fintech sector towards environmentally responsible growth. These combined efforts contribute to the fintech industry's sustainable expansion, bolstering economic activities and pollution.

The overall amount invested in environmental protection in European nations is directed by green finance, and current study prove that green funding takes a detrimental impact on energy shortage. Energy availability may be achieved both directly and indirectly with the use of green financing. Less conventional energy will be used as a result of the rise in green financing. Also, the expansion of green finance will greatly lessen environmental problems. The advanced technologies that minimize industrial pollution are funded by European nations. Swapping out polluting technology with environmentally friendly equipment led to environmental sustainability. Furthermore, the execution of renewable energy projects via green finance helps to lessen environmental issues and energy poverty (H. Zhang et al., 2022). The results indicated a negative correlation between energy poverty and energy efficiency.

Increasing finances and fintech in efficient use of energy can assist to solve the problem of energy poverty. Energy efficiency is one of the key factors that may contribute to improving the energy portfolio. The R&D variable significantly correlates negatively with energy poverty. R&D investment is used in European nations for environmental and energy-related research and development. Although FDI has a favorable influence on the energy sector. Consequently, the bulk of FDI is directed toward businesses that contribute to pollution and are seen as dirty industries, which exacerbates environmental problems and has a negative impact on energy poverty. Energy poverty has a positive relationship with gross domestic product per person. European nations are classified as having highly developed economies, where the average income is greater. Because European nations have touched a high level of development, where an increase in per capita income has led to an improvement in energy efficiency and a reduction in energy poverty through the use of green technologies in industries, their governments place a greater emphasis on environmental issues alongside economic development. Moreover, EU nations have cutting-edge technology, strong environmental regulations, public awareness campaigns, and modern energy systems.

## 4 Conclusion and policy recommendations

The current research analyzed the responsibility of fintech industry, energy efficiency, green finance, R&D, FDI and GDP on energy poverty across European countries from the year 2013–2020. Data availability was a major factor in choosing the European nations and the time series period, particularly the investment data that is now accessible to the fintech sector. Methods of dynamic ordinary least squares (DOLS) and fully modified ordinary least squares (FMOLS) was employed to estimate long run and short run relationships. The study showed that the fintech has significant results with variable of energy poverty. The empirical analysis of the study showed that the investment in fintech industry reduce energy poverty in European countries. Fintech helps to reduce energy poverty and policies of European countries. Because of facilitation of fintech investment, industries’ edition of machines that are sustainable, green production models, equipments that use less energy, green business models help to save energy and reduce energy poverty, significantly.

European countries invest funds in advanced technologies which reduce pollution of industrial sector. The upgradation of pollution intensive equipment with green machinery led to sustainable environment. In addition, installation of renewable energy projects thorough green finance helps to reduce energy poverty. The R&D also has significant and negative impact on energy poverty. Since European nations have advanced development to a certain degree, the GDP per capita also has a favorable effect on energy poverty. Energy security benefits from green financing, and energy poverty, a pressing environmental concern, is mitigated by it. However foreign direct investments (FDI) can alleviate energy poverty.

### 4.1 This study suggests the following implications on the basis of the results

Fintech is significant factor in improving energy portfolio, EU government should promote green investment in fintech by regularization of polices for fintech industry. Investment in R&D is required for reducing energy consumption by advanced level technologies in fintech Industry. There is need for European countries to develop strong polices for high energy intensive industrial sector. Energy poverty may be effectively combated with the support of policies aiming at enhancing the financial standing of vulnerable populations, lowering energy costs, and/or energy efficiency measures. On the other hand, the findings show that metropolitan regions experience worsening energy poverty, which may be a sign of hidden energy poverty in major cities.

Lastly, take notice that this research was conducted at an aggregate level, which has the benefit of enabling comparisons across the various European nations. At this point, it is impossible to pinpoint the precise reasons why disadvantaged populations lack access to energy services. Yet, it is also important to highlight that the model may be used at the microeconomic level to assess causes and policies implemented at the state, provincial, or even municipal level, despite the fact that the research was conducted at the macroscale. In this way, it is feasible to evaluate an area or locale in relation to others throughout a nation. More precise results may be drawn thanks to the availability of more comprehensive data on household and individual variables, including income, degree of urbanization, quality, and type of housing, among others.

The study's policy recommendations are insightful, but certain limitations should be considered. The research was conducted at an aggregated level, making it essential to acknowledge that tailored policies might be needed at more localized levels. Additionally, while the study reveals correlations, it doesn't pinpoint the root causes of energy poverty, necessitating more granular data. The worsening energy poverty in metropolitan areas underscores the need for specific urban-focused analysis. Data limitations, particularly in the fintech industry, highlight the importance of expanding datasets. Lastly, while the recommendations apply to the EU, their generalizability to other regions may be limited due to varying energy poverty challenges and policy requirements.

## Ethics permission and consent to participate

We verified the fact that this paper is original and not currently pending publication in another journal. This research is exempt from informed consent requirements and ethical clearance.

## Consent for publication

Not applicable.

## Funding

Shenzhen Philosophy and Social Science Planning Project Management, SZ2021B010.

## Data availability statement

All data are fully available without restriction. The dataset is taken from a public repository, https://databank.worldbank.org/source/world-development-indicators. http://ec.europa.eu/eurostat.

## CRediT authorship contribution statement

**Zeenat Zia:** Investigation, Formal analysis, Conceptualization. **Ruoyu Zhong:** Writing – review & editing, Writing – original draft, Supervision. **Muhammad Waqas Akbar:** Writing – original draft, Investigation, Data curation, Conceptualization.

## Declaration of competing interest

The authors declare that they have no known competing financial interests or personal relationships that could have appeared to influence the work reported in this paper.
